# Correction: MSP-tracker: A versatile vesicle tracking software tool used to reveal the spatial control of polarized secretion in *Drosophila* epithelial cells

**DOI:** 10.1371/journal.pbio.3003343

**Published:** 2025-08-19

**Authors:** The PLOS Biology Staff

## Notice of Republication

This article was republished on July 24, 2025, to correct an error in the affiliations of the fifth author Richard Butler and in the caption for Fig 6. Please see the complete, correct Fig 6 caption here. The correct affiliation for the fifth author is: ‘The Gurdon Institute and the Department of Genetics, University of Cambridge, Cambridge, UK.’ The publisher apologizes for the errors. Please download this article again to view the correct version.

**Fig 6.** Analysis of Ndg tracks reveals planar polarized transport basally. **A)** Tracks from a representative movie of Ndg trafficking in a stage 8 egg chamber 23 min after biotin addition. The polar plot on the right represents data from 3 Airyscan movies and shows a weak bias towards the basal side of the cell. Speeds are color-coded according to the key. Movies were taken at 3.7 fps. Arrows superimposed on movie stills indicate the vectors of the vesicle tracks from the movie. Cyan arrows are apically directed, red arrows basally directed and yellow arrows laterally directed. **B)** Graph showing the net displacement of Ndg vesicle tracks along the apical-basal axis (A, B; lefthand column) and along the leading edge to trailing edge axis (L,T) in the middle of the cell (middle column) and on the basal side of the cell (righthand column). The bars and error bars show the means and s.e.m. Individual tracks are indicated by the small circles. P indicates planar. Mean displacement towards basal is 0.17 µm ± 0.09 (s.e.m). The mean displacement for planar polarized tracks in the middle of the cell was 0.11 µm ± 0.07 (s.e.m) towards the leading edge, and for the basal tracks, 0.44 µm ± 0.13 (s.e.m) towards the leading edge. Significance was calculated using a Wilcoxon Signed-Rank test comparing the displacement to zero. **C)** Ndg tracks in a transverse section through the middle of the follicle cell epithelium (see diagram). The large white arrow indicates the direction of egg chamber rotation and the coloured arrows indicate the vectors of vesicle tracks, with tracks towards the leading edge in cyan, towards the trailing edge in red and tracks perpendicular to the axis of rotation in yellow. The polar plot on the right represents data from 3 Airyscan movies taken at 2.3 fps. The leading and trailing (L and T) directions of the egg chamber’s rotation are indicated. **D)** Ndg tracks in a transverse section through the basal section of the follicle cell epithelium. The direction of rotation and the tracks are labeled as in (C). Scale bars 5 µm. The polar plot represents data from 3 Airyscan movies taken at 2.3 fps. Track data can be found in S3 Data. Movies used for tracking can be found at https://doi.org/10.17863/CAM.114338.2.

https://doi.org/10.1371/journal.pbio.3003099.g006

The originally published, uncorrected article and the republished, corrected article are provided here for reference.

## Supporting information

S1 File Originally published, uncorrected article. (PDF)

S2 File Republished, corrected article. (PDF)
