# Supplementary figures and images for: MSP-tracker: A versatile vesicle tracking software tool used to reveal the spatial control of polarized secretion in Drosophila epithelial cells
Source: PLoS Biol. 2025 Apr 10;23(4):e3003099. doi: 10.1371/journal.pbio.3003099 (PMC12021295; doi:10.1371/journal.pbio.3003099)

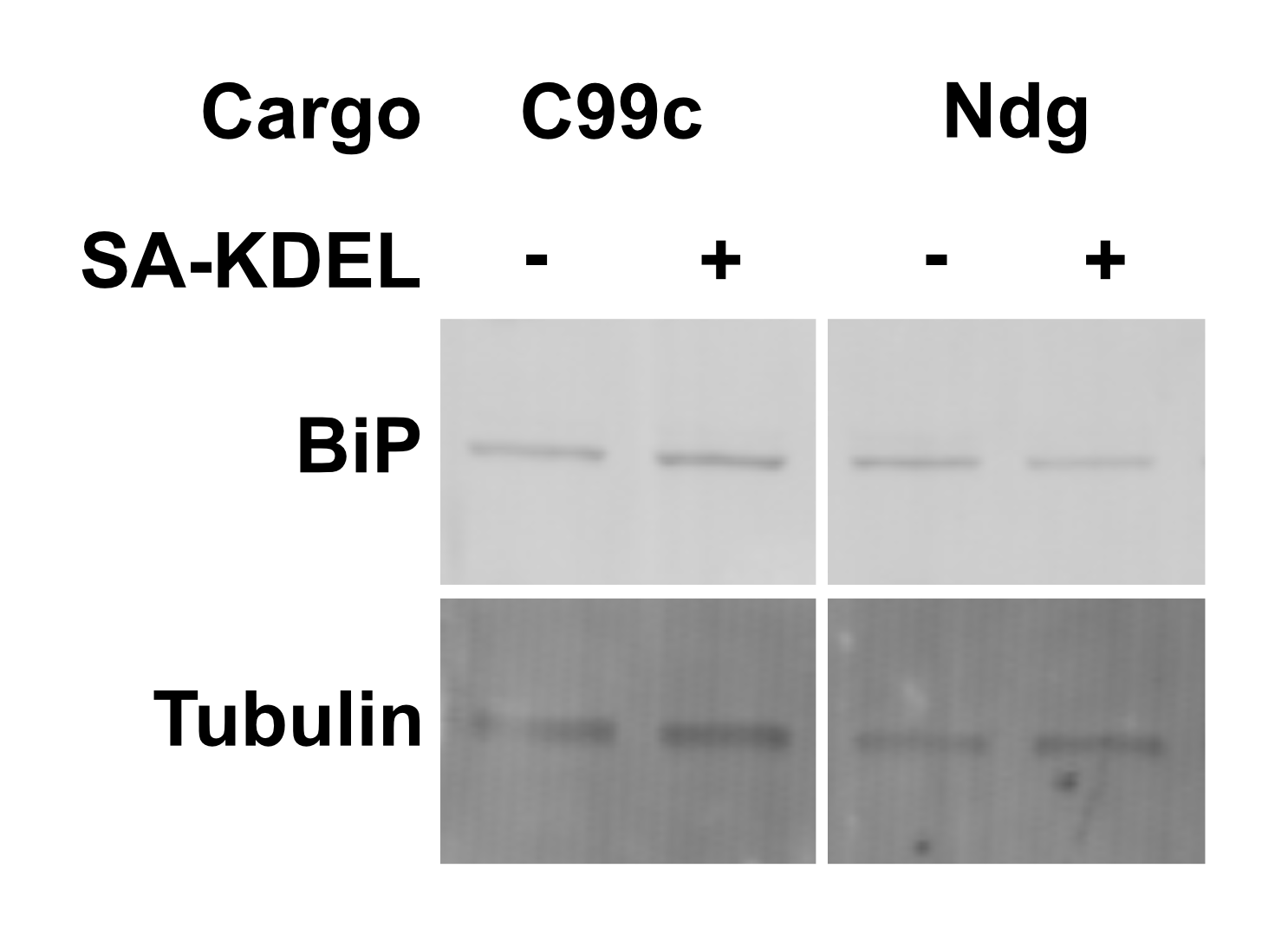

Supplement: S1 Fig — BiP/GRP78 expression is increased when the ER experiences stress, so we measured expression in egg chambers expressing the Cad99c and Ndg RUSH constructs, with or without the 2x Streptavidin-KDEL insertions that retain the cargoes in the ER. Tubulin was used as a loading control. BiP/GRP78 levels do not change when the 2x Streptavidin-KDEL hook was expressed to retain the cargoes in the ER, indicating that trapping the cargoes does not cause ER stress. (TIFF) [file pbio.3003099.s001.tiff]

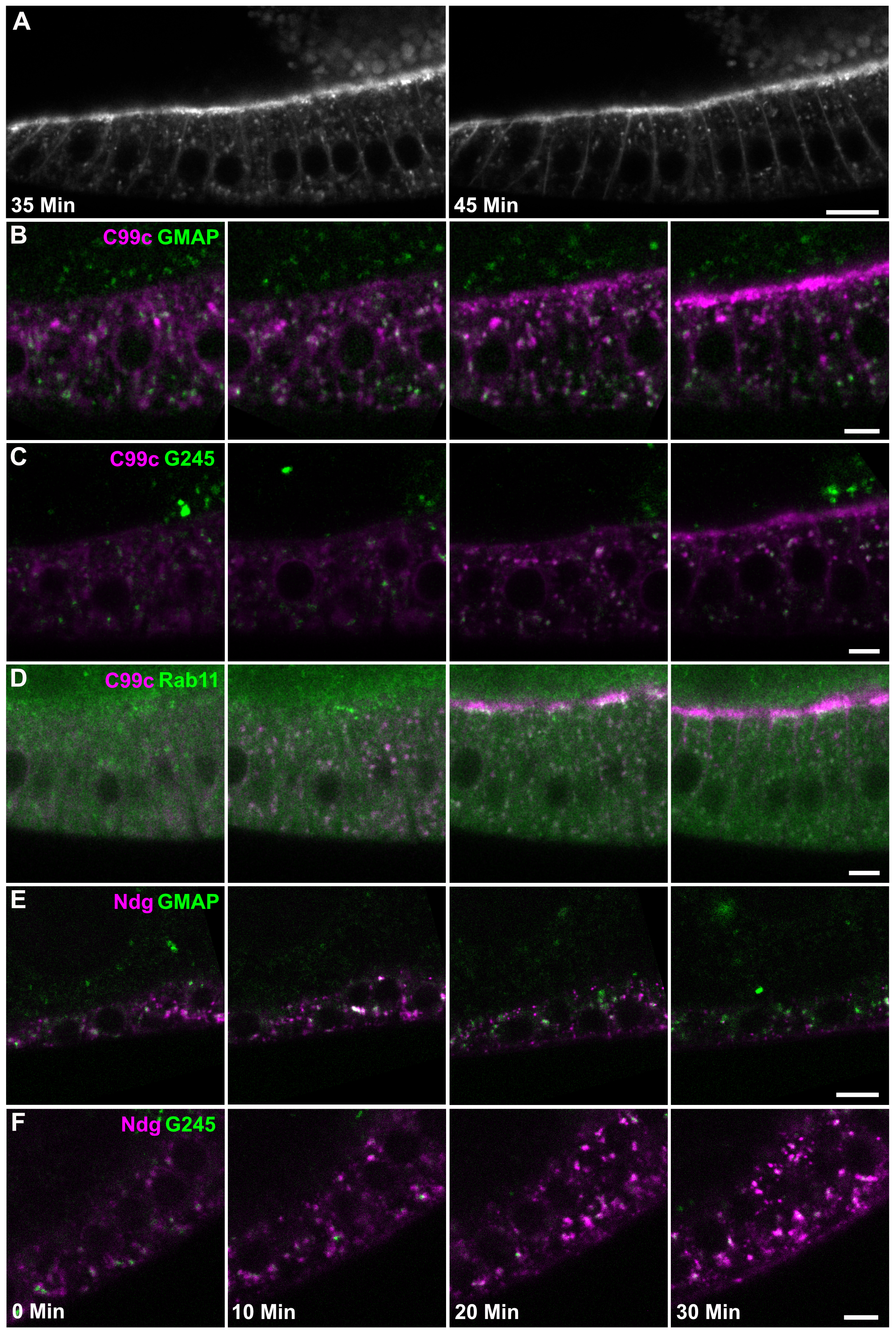

Supplement: S2 Fig — A) Stills from RUSH movies of SBP-Halo-Cadherin 99c trafficking in the follicle cells after release from the ER. Cad99c is shown in an early stage 9 egg chamber. Time indicates minutes since the addition of biotin. Cad99c is labeled with Halo-OregonGreen, At 35 min a small amount of lateral membrane signal can now be seen, which is more pronounced at 45 min. Scale bars 10 µm. B,C&D) Still images taken at different time points are biotin addition from a RUSH movie of SBP-Halo-Cadherin 99c (magenta) trafficking in cells expressing B) GFP-GMAP (cis-Golgi), C) GFP-Golgin245 (trans-Golgi and TGN) and D) YFP-Rab11 (TGN and RE) as secretory pathway markers. Scale bars 5 µm. E&F) Stills from a RUSH movie of SBP-SNAP-Nidogen trafficking in cells expressing E) GFP-GMAP, or F) GFP-Golgin245. Scale bars 5 µm. (TIFF) [file pbio.3003099.s002.tiff]

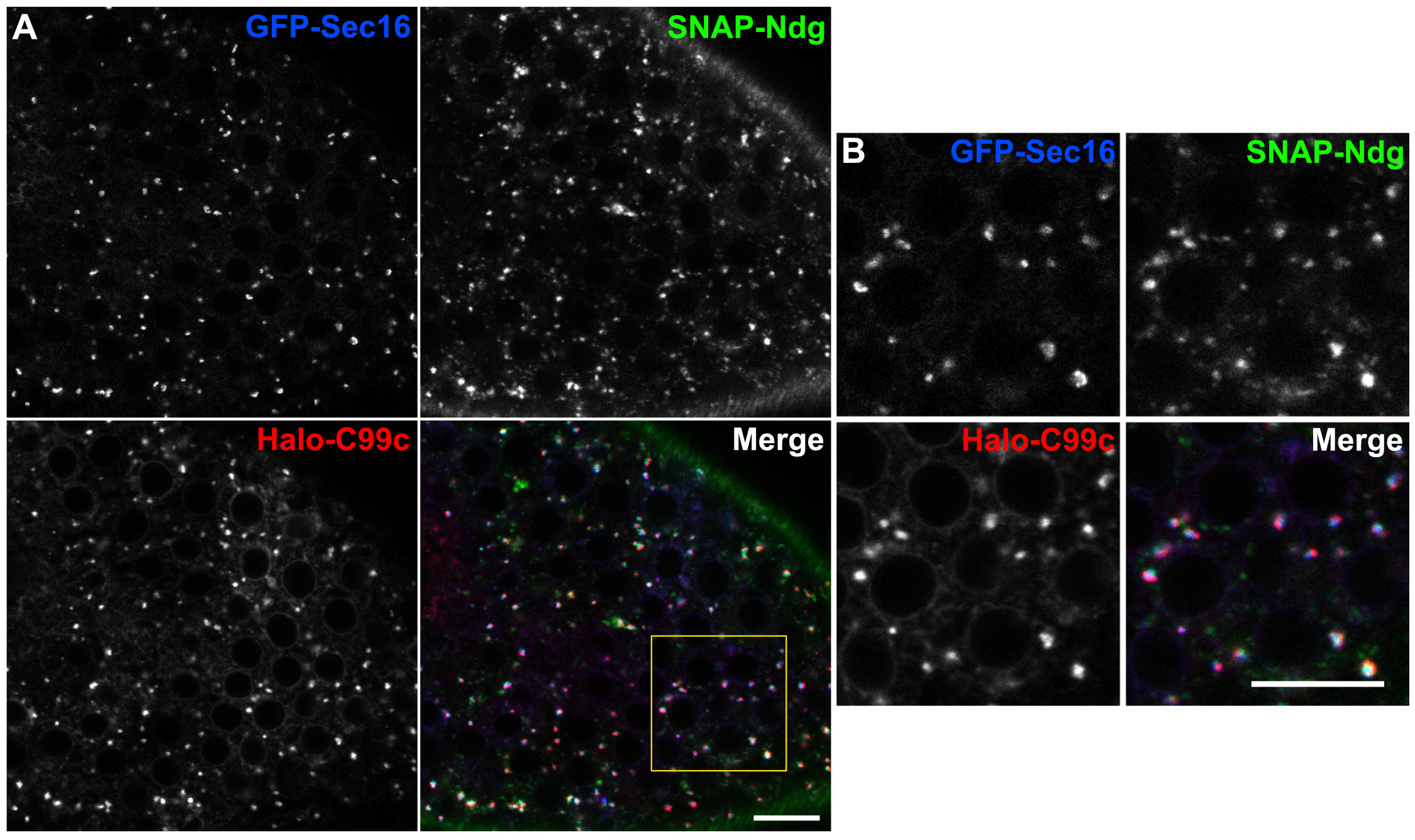

Supplement: S3 Fig — A) Fixed images of an egg chamber expressing both SBP-SNAP-Ndg and SBP-Halo-Cad99c in the RUSH system. Egg chambers were fixed after 5 min in biotin, to allow RUSH to proceed as far as the ER exit sites and cis-Golgi. SNAP-Ndg was labeled with SNAP-SiR dye, and Halo-C99c with Halo-JF549. ER exit sites were marked by the presence of GFP-Sec16. B) shows a zoomed in view of the yellow boxed area in A) showing that both Ndg and Cad99c are present at all the ERES that are labeled with GFP-Sec16 (endogenously tagged). Scale bars 10 um. (TIFF) [file pbio.3003099.s003.tiff]

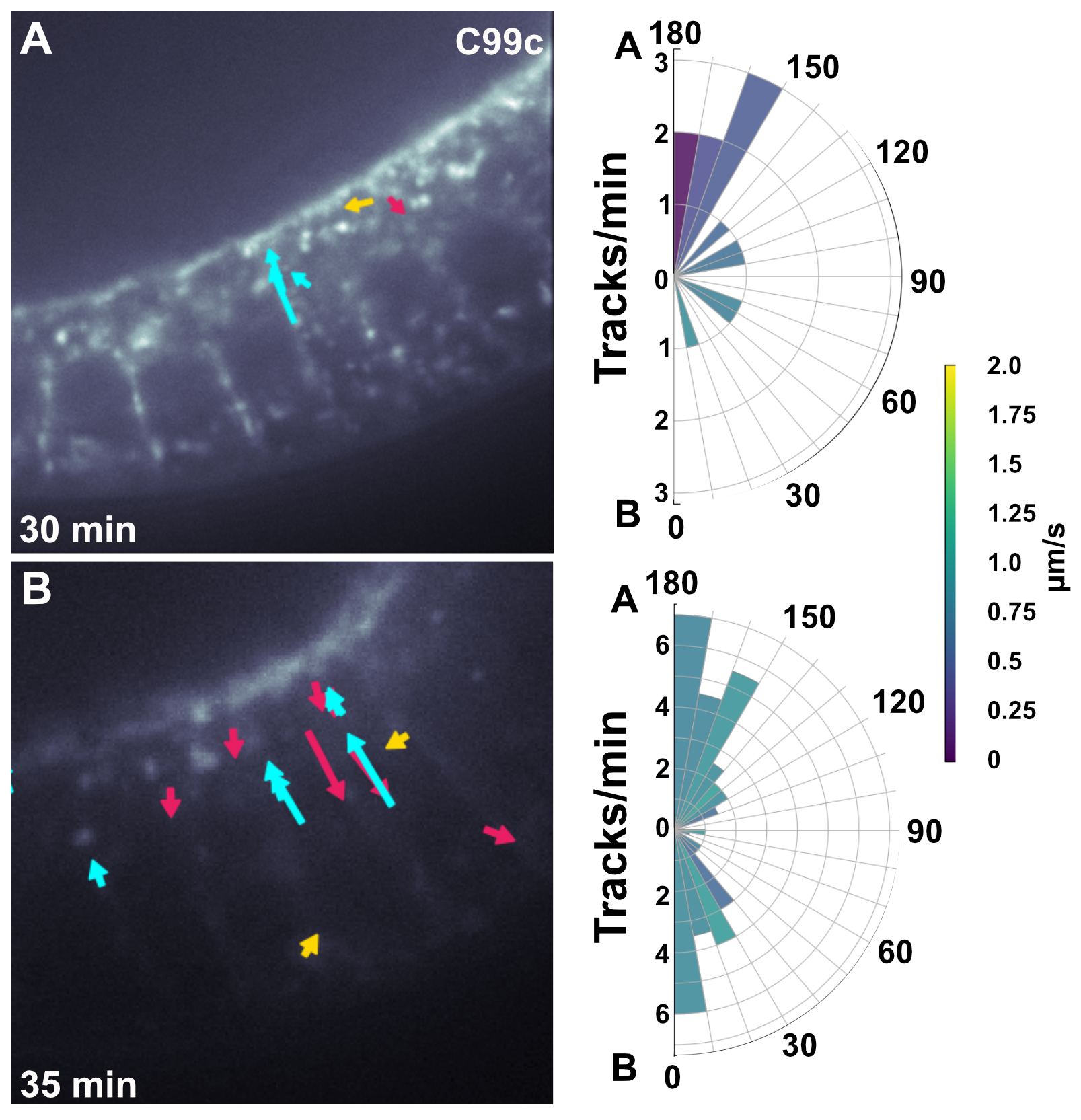

Supplement: S4 Fig — A) Tracks from representative movie taken on a spinning disc microscope of Cad99c trafficking in a stage 9 egg chamber, 30 min after biotin addition. The polar plot on the right shows the strong apical bias in the direction of the tracks. Movies were taken at 4 fps using a custom-built spinning disc system. B) Tracks from a representative movie taken on a spinning disc microscope of Cad99c trafficking in a stage 9 egg chamber, 35 min after biotin addition. The polar plot on the right represents data from 2 spinning disc movies and shows that the strong apical bias has disappeared, as the exocytosis of C99c is now balanced by endocytosis. Track data can be found in S4 Data. Movies used for tracking can be found at https://doi.org/10.17863/CAM.114338.2. (TIFF) [file pbio.3003099.s004.tiff]

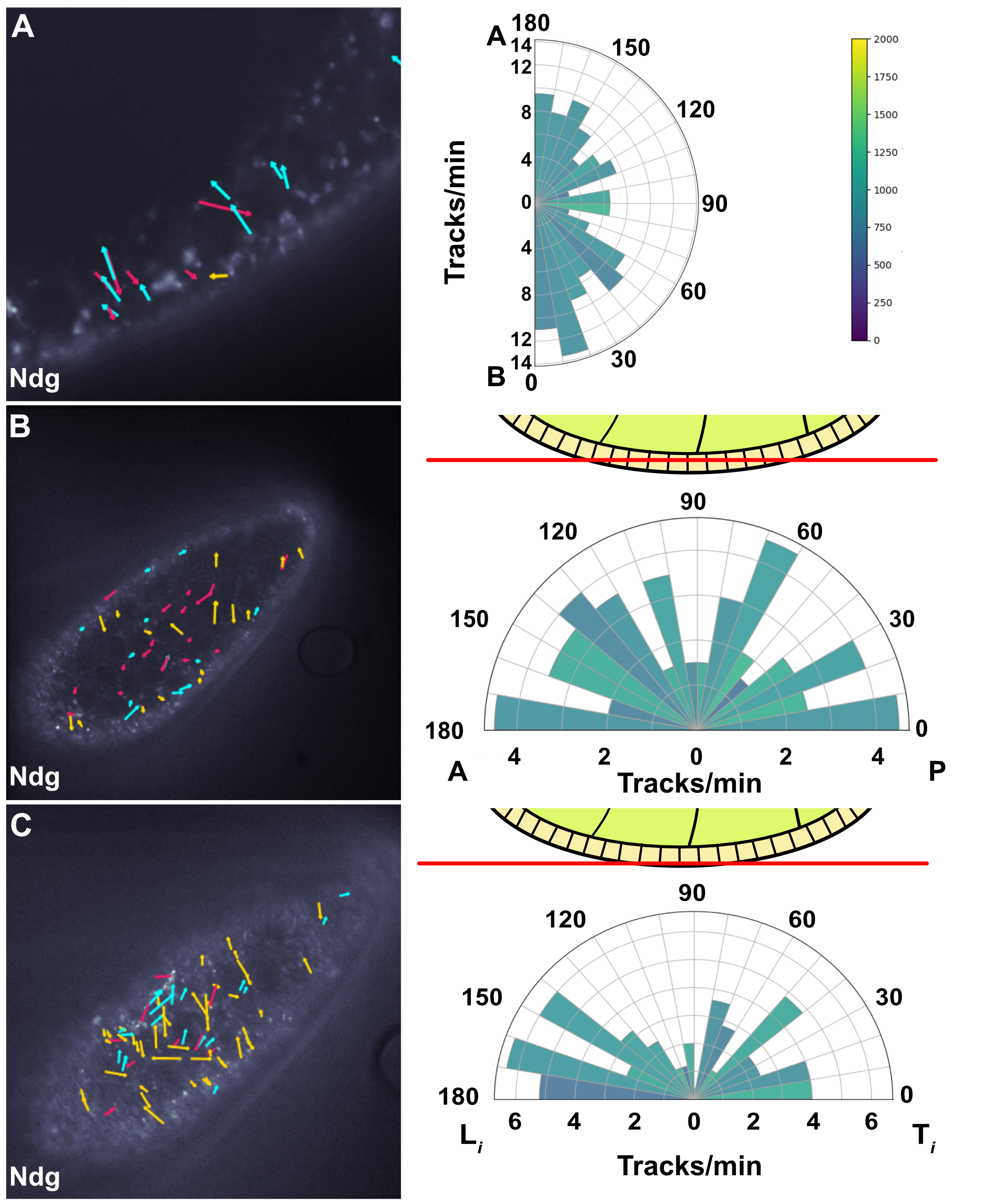

Supplement: S5 Fig — A) Tracks from representative movie taken on a spinning disc microscope of Ndg vesicle movements along the apical-basal axis of a stage 8 egg chamber. The polar plot on the right represents data from 2 spinning disc movies, and shows a slight basal bias in Ndg movements. B) Tracks from a representative movie taken on a spinning disc microscope of Ndg vesicle movements Ndg tracks in a transverse section through the middle section of the follicle cells (see diagram). The polar plot represents data from 3 spinning disc movies and shows no clear bias in track direction. C) Tracks from a representative movie taken on a spinning disc microscope of Ndg vesicle movements in a transverse section at the basal side of the follicle cells (see diagram). The leading and trailing (L and T) directions of follicle cell migration are indicated in the polar plot on the right, which shows that most tracks are directed towards the leading edge. Track data can be found in S4 Data. Movies were taken at 2 fps. Movies used for tracking can be found at https://doi.org/10.17863/CAM.114338.2. (TIFF) [file pbio.3003099.s005.tiff]

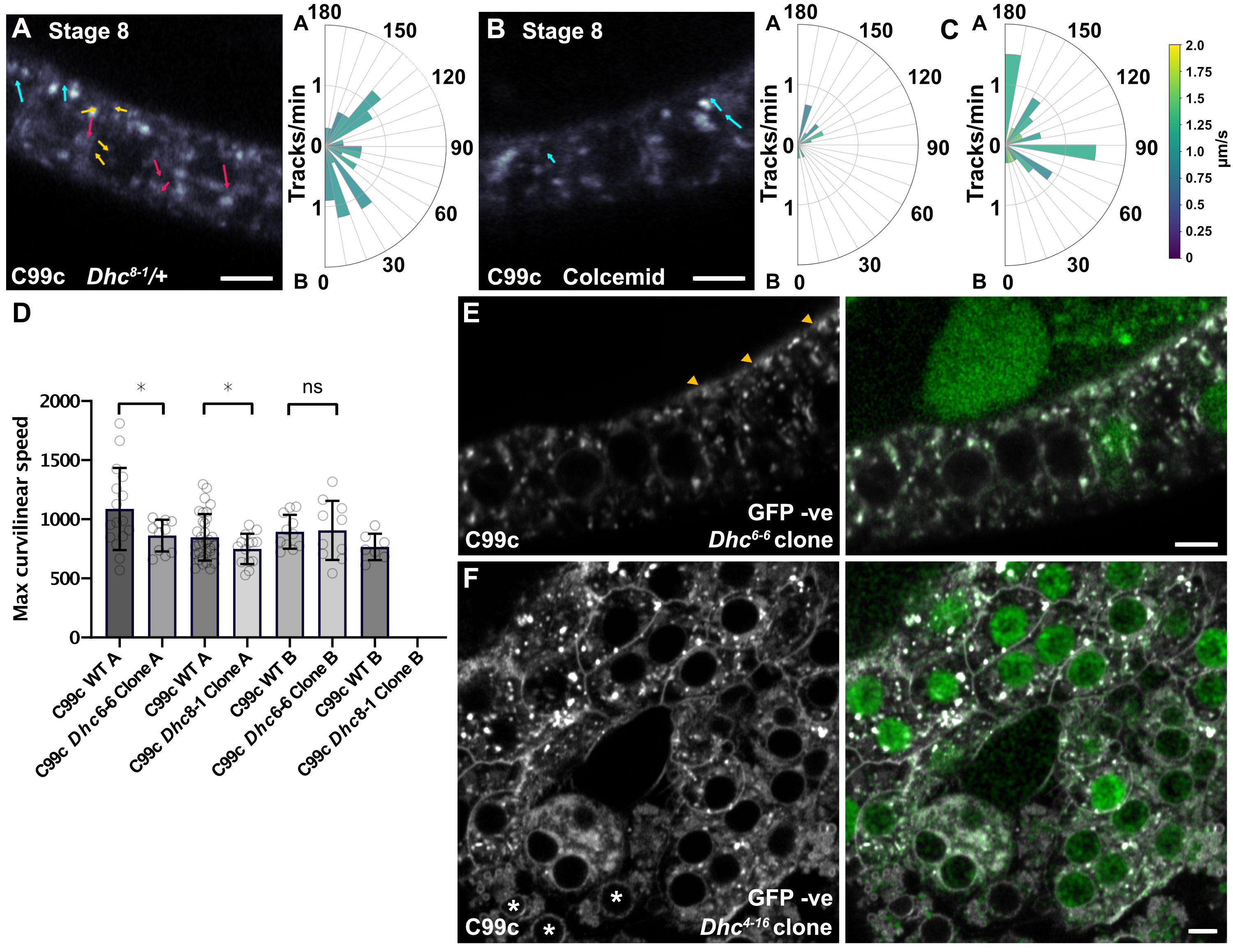

Supplement: S6 Fig — A) Tracks from a representative movie of Cad99c trafficking in a Dhc8-1 heterozygous stage 8 egg chamber 33 min after biotin addition. Scale bar 5 µm. The polar plot on the right represents data from 3 movies. B) Tracks from a representative movie of Cad99c trafficking 24 min after biotin addition in a stage 8 egg chamber that has been treated with colcemid to depolymerize microtubules. Scale bar 5 µm. The polar plot on the right represents data from 4 movies C) Polar plot representing data from 2 movies showing the number of Cad99c tracks per minute in wild-type stage 8 egg chambers. Graph scales in A) B) and C) are the same to allow for easy comparison. D) Graph showing the maximum curvilinear speeds (over a segment) of Cad99c vesicles traveling apically in wild-type and Dhc mutant clones. Cad99c vesicles move significantly faster in wild-type cells than in either the Dhc6-6 or Dhc8-1 homozygous cells. The bars and error bars represent the means and standard deviations and the circles represent individual tracks. Statistical significance was determined with a two-tailed Welch’s T test with significant differences between conditions indicated: * = p < 0.05. E) A still image of Cad99c RUSH in a Dhc6-6 homozygous clone marked by the loss of nuclear GFP (green) 51 min after biotin addition. Cad99c has progressed further through the secretory pathway in the wildtype cells (green nuclei) than in the mutant cells (arrowheads). Scale bar 5 µm. F) A still image of RUSH in Dhc4-16 homozygous clones marked by the loss of nuclear GFP (green) at 26 min after biotin addition. Dhc4-16 is a strong allele of Dhc64c and has caused gaps to form in the epithelium. Cad99c has reached the plasma membrane in the wild-type cells (GFP positive nuclei), whereas it has not reached the membrane in the mutant cells (asterisks). Scale bar 5 µm. Track data can be found in S3 Data. Movies were taken at 4.7 fps. Movies used for tracking can be found at https://doi.org/10.17863/CAM.114338.2. (TIFF [file pbio.3003099.s006.tiff]

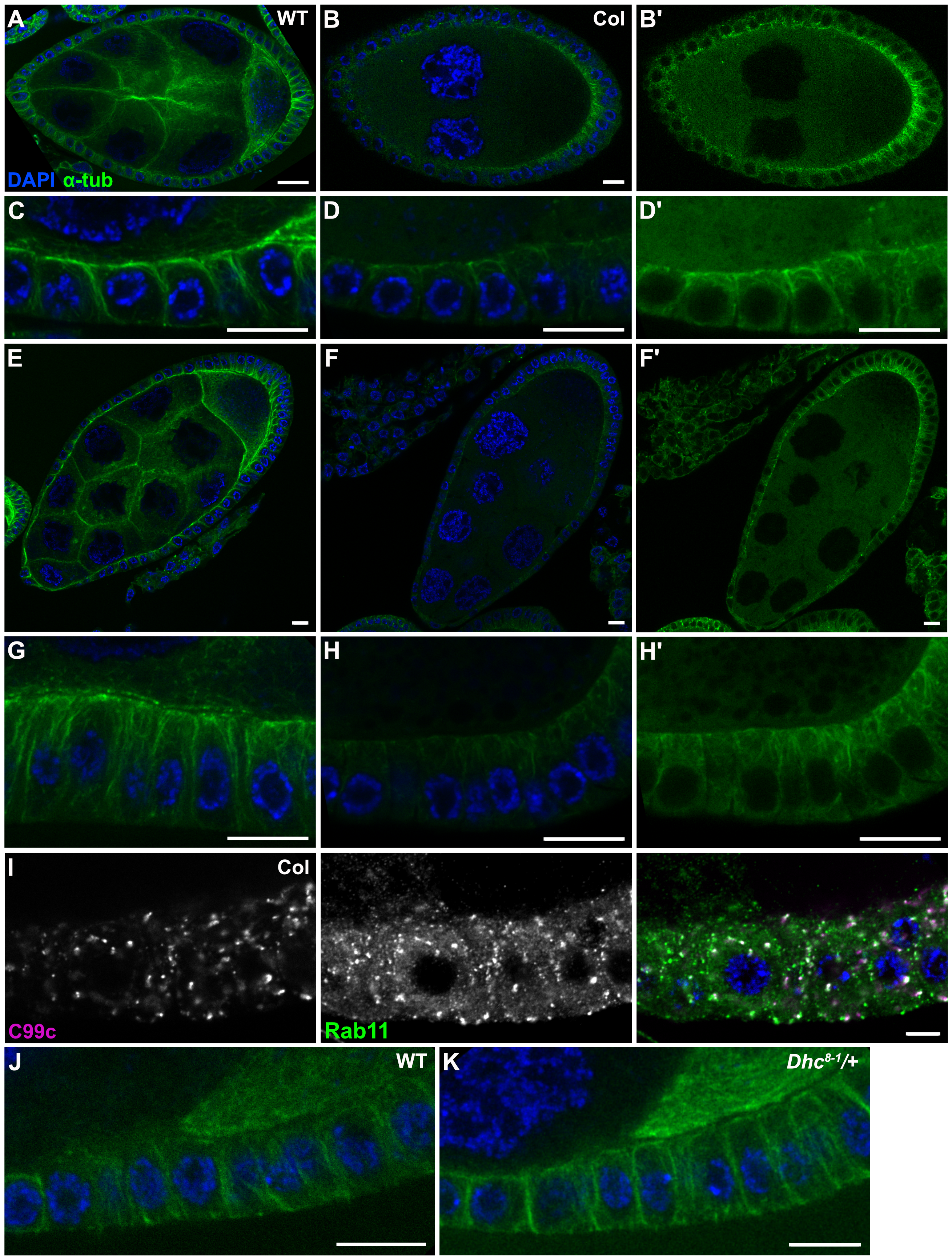

Supplement: S7 Fig — A), C), E), G) β-tubulin staining in mock injected flies. Scale bar 10 µm. B), D), F), H) β-tubulin staining in flies injected with colcemid. These images were collected with the same microscope settings and laser power colour scale as the mock injected images. Scale bar 10 µm. B’), D’), F’), H’) have all been adjusted to show the details of the remaining microtubules. C) and D) show microtubules in cuboidal follicle cells. G) and H) show microtubules in columnar follicle cells. I) Rab11 and Cad99c localization in colcemid-treated egg chambers 30 min after the addition of biotin. Rab11 positive recycling endosomes are no longer enriched apically and can be seen laterally and basally with Cad99c. Scale bar 5 µm. J) β-tubulin staining in yw flies. Scale bar 10 µm. K) β-tubulin staining in Dhc8-1/+ flies. Scale bar 10 µm. (TIFF) [file pbio.3003099.s007.tiff]

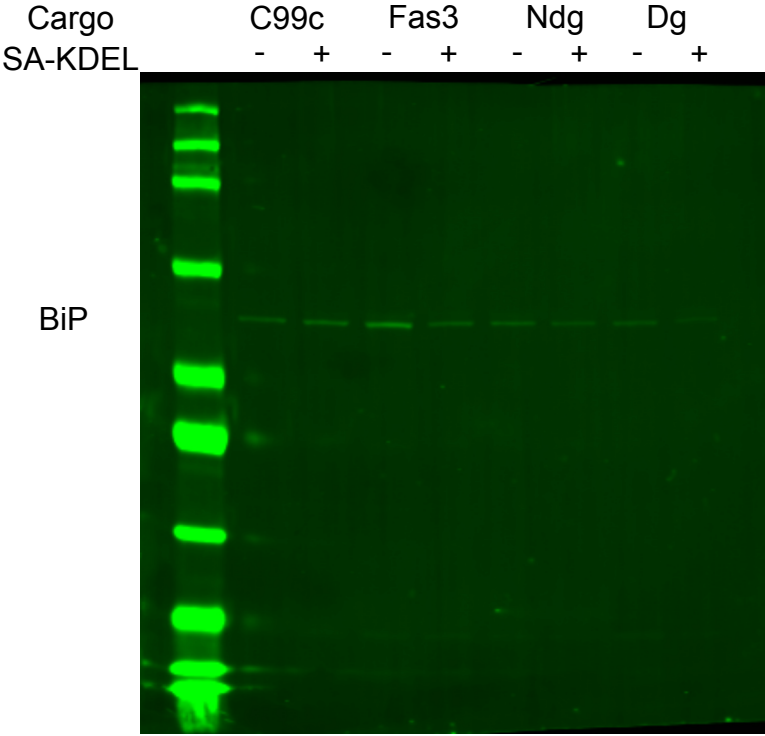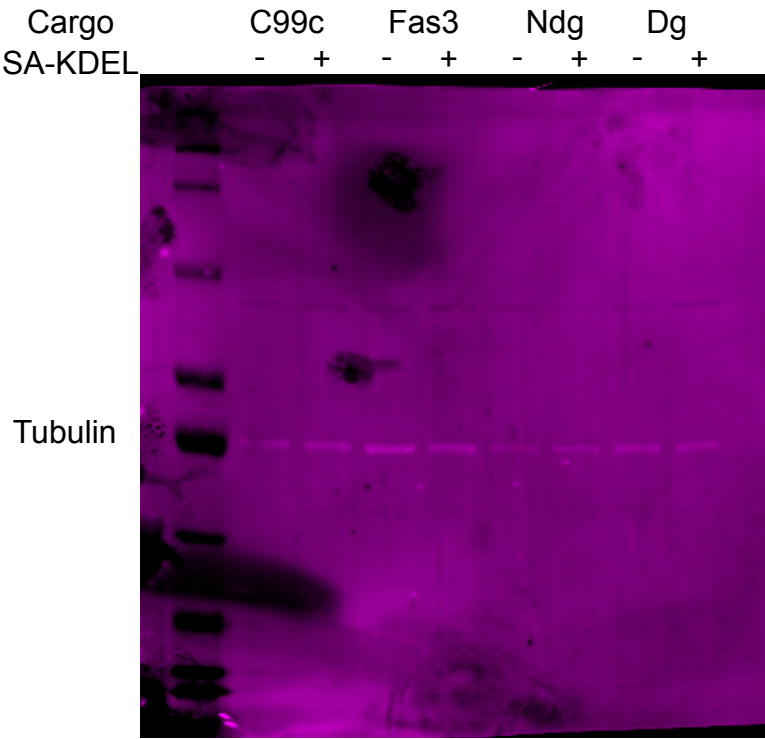

Supplement: S1 Raw Images — (PDF) [file pbio.3003099.s009.pdf]
