# Supplementary material for: MSP-tracker: A versatile vesicle tracking software tool used to reveal the spatial control of polarized secretion in Drosophila epithelial cells
Source: PLoS Biol. 2025 Apr 10;23(4):e3003099. doi: 10.1371/journal.pbio.3003099 (PMC12021295; doi:10.1371/journal.pbio.3003099)
Supplement: Attachment — Submitted filename: Response to reviewers comments.docx [file pbio.3003099.s028.docx]

REVIEWS:

Reviewer #1: In the manuscript by Richens et al., the authors present a significant advancement in understanding the targeting of specific secretory cargoes to distinct domains of the plasma membrane in epithelial cells. Using the classic model system of Drosophila epithelial follicle cells, they apply two innovative methods: the RUSH system and the MSP tracking framework. While I appreciate these groundbreaking methods, I have some major concerns about certain conclusions and hope the authors can address them in their revised manuscript. 
 (1) 1) The MSP tracker is a powerful method, particularly effective for tracking in noisy and dim images due to its machine learning module. However, while following their detailed manual with step-by-step instructions, I encountered persistent error messages in Anaconda when running tests to identify particles, specifically showing "AttributeError: 'str' object has no attribute 'decode'." I speculate that this issue may stem from differences in string function definitions across Python versions (I am using Python 3.11.7 on a Windows 10 computer). Due to the complexity and length of the original code, I was unable to fully review the quality of the MSP tracker. I highly recommend that the authors provide a step-by-step video tutorial to facilitate broader adoption and usage of the MSP tracker.

The reviewer is correct that this problem is caused by differences between Python versions. We have now addressed this issue by building a docker container that keeps the same environment for all users, regardless of their operating system. This can now be found at https://doi.org/10.17863/CAM.114339

Following the referee's suggestion, we have added video tutorials on how to open and use MSP-tracker and MSP-viewer, which can also be found at https://doi.org/10.17863/CAM.114339

(2) The authors used a slower dynein mutant (Dhc[8-1]) to demonstrate that Cad99c apical transport is dependent on dynein. In Dhc[8-1]/+ heterozygous cells, the apical velocity of Cad99c decreased from 1.1 μm/sec to 0.85 μm/sec. I have two concerns regarding this part: (a) Cytoplasmic dynein is known to affect microtubule organization in both dividing cells (mitotic spindle formation) and non-dividing cells (neurons, nurse cells, etc.). Therefore, the authors need to ensure that there is no significant microtubule reorganization in Dhc[8-1]/+ cells

This is a valid point. We have therefore examined the organisation of microtubules in Dhc^8-1^/+ cells and observed no apparent differences from wild-type. These data have now been added as Supplementary Figure 6J and K.

(b) Since the measurement was done in Dhc[8-1]/+ cells, it is difficult to determine whether dynein or another unidentified motor is responsible for the residual apical transport. I recommend that the authors measure Cad99c transport in Dhc[8-1] homozygous FRT clones. A previous paper from the same lab (Reference [58], Vítor Trovisco et al., 2016, eLife) reported the use of homozygous mutant germline clones of Dhc[8-1],
indicating that this dynein mutant does not cause significant cell division defects and thus should allow the production of FRT clones in follicle cells. Alternatively, a trans-heterozygous mutant of Dhc[6-10]/Dhc[8-1] or TRiP RNAi lines targeting Dhc64C could be used.

The Dhc^8-1^ mutant does not inactivate dynein, but makes it move more slowly. This is why there is still apical transport of Cad99c vesicles in Dhc^8-1^/+, but their speed is reduced, whereas the speed of basal movements is unaffected (Figure 8D). This allele has a dominant effect, presumably because dynein functions as a dimer and one mutant copy is sufficient to slow the dimer down. 25% of dimers should contain two wild-type copies of dynein, which may explain the small number of residual fast movements. Nevertheless, as requested, we have now examined Cad99c trafficking in *Dhc*^8-1^ homozygous clones and in clones of *Dhc*^6-6^, which also reduces the speed of dynein movement [1]. In both cases, there is a significant reduction in velocity of Cad99c vesicles towards the apical side of the cell, but no change in the speed of basal movements. We also made clones of a stronger allele, *Dhc*^4-16^, which led to gaps in the epithelium and a complete block to Cad99c secretion. These data have been added to Supplementary Figure 5.

(3) While the role of dynein in transporting Cad99c vesicles to the apical membrane is clear, the section on Ndg transport is less so, and some conclusions are puzzling.
(a) The authors showed that overexpression of Ndg shifts Rab11 directionality towards the leading edge, whereas in wild-type cells, Rab11 is predominantly directed towards the trailing edge. This suggests that Ndg and Collagen IV (and probably other vesicles toward the trailing edge) compete for Rab11 vesicles. This raises the question of whether endogenous Ndg shows a preference for the leading edge. I recommend the authors conduct a similar FRT clone experiment (as was done for the Vkg clone) to determine if endogenously secreted Ndg preferentially goes to the leading edge.

We presume that the referee meant to refer to Rab10 rather than Rab11. We attempted the experiment that they suggested to demonstrate that endogenous Nidogen is secreted from the leading edge. This was not informative because Nidogen is soluble and rapidly diffuses from its site of secretion until it binds to the ECM. One therefore detects as much accumulation between non-expressing follicle cells as between expressing cells.

An image from a movie of an egg chamber containing Ndg-GFP expressing cells (no nls-RFP) and non-expressing cells (nls-RFP; red). The red arrowhead indicates the direction of rotation of the egg chamber. This was an unsuccessful attempt to repeat the experiment that we performed with Vkg-GFP clones, using Ndg- GFP clones. The Ndg-GFP trap line is extremely weak and the GFP signal is hard to distinguish from autofluorescence of the egg chamber. While one can detect weak labelling of the extracellular Ndg with the Atto647n-anti-GFP antibody, as much signal appears

between the non-expressing, nls-RFP positive cells, as between the Ndg-GFP expressing cells. This presumably results from the diffusion of Nidogen away from its site of secretion and its subsequent binding to ECM between the non-expressing follicle

cells.

We also attempted to examine the direction of Collagen IV secretion in cells in which we also released a wave of Nidogen secretion to test whether this would affect the direction of Collagen IV secretion. However, it proved impossible to generate the flies in which we could generate Viking-GFP positive and negative clones that also contained the transgenes necessary for RUSH (traffic jam-Gal4, two copies of the UAS Streptavidin-KDEL hook and UAS- SBP- Nidogen).

We do not envisage that Nidogen and Collagen IV compete for inclusion in Rab10+ve vesicles. Rab 10 is required for the basal transport of ECM cargoes, since these are secreted apically after Rab10 knock down or in mutant clones for the Rab10 GEF, *crag* [2–4]. This suggests that Rab10 is required for the transport of ECM vesicles containing Collagen IV and Nidogen to the basal side of the cell, where they accumulate into Rab10^+ve^ punctae near the trailing edge[4]. Rab10 might then be a passenger on the vesicles that leave these punctae to move either to the leading edge (most Nidogen) or trailing edge (most Collagen IV).

(b) It is puzzling that the authors suggest dynein is not required for the apical transport of Ndg vesicles. Given that the microtubule network is highly polarized, with minus ends near the apical side and plus ends extending towards the basal side, do the authors suggest another minus-end-directed motor is involved? Alternatively, it is possible that Ndg requires less active dynein for apical transport, making the effect not significant in Dhc[8-1] heterozygous cells.

The evidence that dynein is not required for the apical movements of Nidogen vesicles is that their speed is not affected by the slow dynein mutant, *Dhc*^8-1^. It is possible that this movement is mediated by dynein, but by a different form of the dynein motor complex that is not affected by the *Dhc*^8-1^ mutation. We have therefore amended the text as follows: "*In contrast to Cad99c, the apical movements of Nidogen-containing vesicles were unaffected by the Dhc^8-1^ mutant, suggesting that their trafficking either does not require Dynein, or is mediated by a form of the Dynein motor complex whose speed is unaffected by the 8-1 mutation, which has not been molecularly characterised.*" It is not the case, however, that Nidogen could be transported apically by less active dynein, since it actually moves faster than Cad99c (1.5µm/sec versus 1.1µm/sec).

The authors further suggest that Khc-73 is required for the basal transport of Ndg vesicles. This hypothesis could be tested by combining the Khc-73 mutant with the RUSH method and MSP-tracker.

The evidence for this comes from Zajac et al, 2022 who showed that Collagen IV is secreted at the top of the lateral domain in Khc-73 knock down cells. Since Khc-73 is a fast motor and we see Nidogen vesicles moving basally at an average speed of 1.5 µm/sec, it seems reasonable to propose that Khc-73 is transporting Nidogen vesicles basally. We are currently generating the stocks to test this by knocking down Khc-73 with RNAi, but think that this is beyond the scope of this manuscript.

(c) The authors argue that "transport towards both the leading and trailing edges is mediated by plus-end-directed motors antagonized by dynein." However, microtubules on the planar plane are highly polarized, extending from the leading edge to the trailing edge with their minus ends at the trailing edge.

Tracking EB1 comets has shown that microtubules grow in both directions. Viktorinova and Dahmann (2013) reported that 75% of microtubules grow towards the trailing edge and 25% towards the leading edge, while Zajac et al. (2022) reported that 84% grow towards the trailing edge [4,5]. Nevertheless, we agree that the increase in speed of these movements in *Dhc*^8-1^/+ does not provide strong evidence that both these movements are mediated by plus end directed motors. We have therefore amended the text as follows: "*This suggests that dynein does not mediate transport towards the leading or trailing edge, although why these movements are faster is unclear.*"

I find it difficult to understand how plus-end-directed motors could mediate movement in both directions along such a polarized network. Based on velocity and previous publications, the authors suggest kinesin-1 is responsible for these movements, although the planar Ndg velocity (~0.7 μm/sec) is on the higher end of the reported kinesin-1 velocity (~0.4 μm/sec). The involvement of kinesin-1 could be tested using well-established kinesin-1 mutants.

We are knocking down various kinesins to test whether they play any role in planar-polarised Nidogen transport, but again think that this is beyond the scope of this work.

Additionally, at the end of the results section, the authors suggest that "vesicle movements towards the leading edge are significantly faster than those towards the trailing edge in Dhc[8-1]/+, suggesting that different motors move the vesicles in each direction." However, Figure 8D indicates not significant ("ns") between the last two sets of data."

We agree with this point and have removed this comment.
 
Minor revision points:
1. For supplemental figures, the figure panels are not referred to sequentially in the text. Additionally, they should be renamed as "Supplement for Figure X" (as in the figure legend) instead of "Figure Sx" (as in the text). Otherwise, it is confusing that only Figure S1, S2, S5, S6, and S8 are mentioned.

We have renamed the Supplementary Figures following the PLoS Biology format, i.e Supplementary Figure X (related to Figure Y).

We apologise for this mistake and have corrected it.

2. The overexpression patterns of Cad99c and Ndg were not shown. If possible, please provide antibody staining to show the endogenous localization of Cad99c and Ndg for readers to compare.

We have now added panels showing the steady state expression of UAS-SBP-Halo-Cadherin99c and UAS-SBP-SNAP-Nidogen under the control of traffic jam-Gal4 as Figure 1 C-E. The endogenous localisation of Cad99c has been published by Khanal et al., 2016 (Figure 1D) and Schlichting et al., 2005, (Figures 4 and 5) and that of Nidogen in Dai et al., 2018,( Figure 1F) and we have now added these references to the text.

On page 4, Figure 1C is referred to incorrectly: "although some protein leaks into the lateral domain because of overexpression (Figure 1C)". Figure 1C does not match this text description. Later on page 5, Figure 1C is referred to again (this time correctly): "On the addition of biotin, it is released and most Cad99c is localized in the Golgi after 15 minutes (Figure 1C)".

We have corrected this mistake and now show the leakage of Cad99c at later time points in Supplementary figure 2A. We have amended the text as follows: "*At later time points, Cad99c often spread into the lateral membrane (Supplementary Figure 2A), probably because the over-expressed protein saturated its apical binding sites and diffused in the plasma membrane after secretion.*"

3. On page 5: "Expressing two copies of UAS-Streptavidin-KDEL is sufficient to retain all SBP-Halo-Cad99C in the ER without activating the ER stress response, as monitored by the levels of BiP [39] (Figure S1)." Throughout the manuscript, BiP was never properly introduced. Please include a brief introduction to BiP.

We have now amended the text as follows: "*Expressing two copies of UAS-Streptavidin-KDEL is sufficient to retain all SBP-Halo-Cad99C in the ER without activating the ER stress response, as monitored by the levels of BiP, a molecular chaperone that is up-regulated in response to ER stress*[6]".

4. On page 5, regarding Rab11 recycling endosomes: "Cad99c co-localizes with Rab11 in puncta immediately below the apical membrane before it reaches the apical surface, indicating that it passes through Rab11-positive recycling endosomes en route to the cell surface (Figure 1D)." Does a dominant negative Rab11 mutant affect Cad99c reaching the apical microvilli?

This experiment has already been performed by Khanal et al. (2016), who showed that RNAi mediated knock down of Rab11 blocks the apical localisation of Cadherin 99c. We have now added this reference.

5. For Figures 2G and 2H, what are the sample sizes for each quantification? SEM or 95% CI are needed on these curves to show the range for each time point.

All sample sizes are in Table 3 in the Materials and Methods. We did not include the SEM in the figures because they obscure the headline results, but we have now replaced these panels with versions that include the SEM.

6. Do the follicle cells rotate in Movie S6? If not, how are the leading edge versus trailing edge defined?

Yes, the follicle cells move a short distance over the 2 minutes of movie 6C, revealing the direction of rotation. This is most obvious if you rapidly drag the time slider from the start to finish of the movie.

7. On page 11: "This increase in directionality correlates with the more highly polarized organization of the microtubules along the apical-basal axis of columnar follicle cells compared to the cuboidal cells at stage 8 (Figure S8G and K)." It is difficult to determine which example has more polarized MTs. Some quantitative measurement is needed here. Alternatively, the authors could use a more direct trackable marker, such as EB1, to show a more polarized MT network at stage 9.

We do not have the time or resources to carry out the experiments that the reviewer is asking for, so we have rephrased this sentence as follows" *This increase in directionality correlates with the increase in the abundance of long microtubules that extend along the apical-basal axis of the columnar follicle cells compared to the cuboidal cells at stage 8 (Fig S8G and K)* [7–9]"

8.  In Figure 7G, it is hard to discern whether there is a bias of extracellular Viking-GFP accumulation at the trailing edge. Please add arrowheads to make it clearer where to look.

Done

9. On page 14: "Colcemid treatment leads to the depolymerization of almost all microtubules in the follicle cells, but a few stable microtubules persist in the apical region of the cells (Figure S8)." Figure S8 is large and complex. It should be more specific; please refer to Figure S8F, H, J, L.

Done

10. On page 15, at the beginning of the discussion: "[20] The trafficking of Cad99C to the apical surface of the follicle cells has been shown to depend on dynein and microtubules in studies in fixed samples [52]." It is unclear why reference [20] is placed at the beginning of the discussion.
We apologise for this error.


Reviewer #2: This manuscript by Richens et al. describes a study of polarized secretory traffic in the Drosophila follicular epithelium. The researchers employ the RUSH technology, previously implemented for in vivo/in animal studies in Drosophila (Glashauser et al., 2023), to create waves of two secretory cargos and study in live recordings their progress through the secretory pathway towards the plasma membrane. The first of these two experimental cargos is an apically localized transmembrane adhesion protein (Cad99C), the second one a basally localized ECM protein (Nidogen). For the quantitative analysis of the live recordings of the two cargos, the manuscript describes the development of a tracking tool called MSP-tracker that allows them to quantitate cis-trans progress with respect to secretory pathway markers, as well as direction and speed within the cell. Based on these data, Richens et al show that post-Golgi movement of each cargo displays a directional bias:
apical for Cad99C, basal for Nidogen. In addition, after analysis of dynein mutants and colcemid treatments, the authors make points of some novelty on how the two cargos are moved within the cell and targeted to their place of secretion: in the case of Cad99C, dynein mutation and colcemid treatment abolish apical bias (in number of apically-directed tracks and their higher speed), which results in basolateral secretion; basally biased movement of Nidogen, in contrast, is independent of dynein, but, interestingly, its whole post-Golgi movement depends on microtubules, as colcemid abolishes it completely. A major point of interest extracted from the data is that Nidogen basal secretion is planarly polarized towards the leading edge of the migrating follicle cells, which is in contrast to Collagen IV, another ECM protein, previously shown to be targeted through kinesin transport to the opposite, trailing end (Zajac and Horne-Badovinac, 2022).

I support publication of this manuscript provided several points are addressed. These hopefully do not involve extensive new experiments, but additional analysis of existing data, discussion, re-interpretation and rewriting.

1. A point central to the spatial control of secretion the manuscript is trying to address is whether ERES are all the same or specific in what they secrete. Could the authors extract from their movies the answer to this question by comparing the spatial distribution of the ERES-Golgi origins of the two cargos?

We have examined follicle cells co-expressing SBP-Halo-Cadherin99c and SBP-SNAP-Nidogen after their release from the ER, while labelling ER exit sites with GFP-Sec 16. As the accompanying Figure shows, both proteins appear to pass through all ERES. We have not included these data in the manuscript because they are tangential to its main subject.

2. The authors state that Cad99C is secreted apically through Rab11 endosomes. This was also a conclusion in Khanal et al., 2016. Indeed, Rab11 in Fig 1D shows colocalization with Cad99C in cytoplasm and both are enriched sub-apically. However, Rab11 resides in most Golgi, as reported recently in other Drosophila cell types (Zhou et al., 2023). Therefore, it would be important to know whether Rab11 and Cad99C track together from trans-Golgi to apical PM. The actual movie (Movie 1 does not show Rab11) and a colocalization plot like the one for Rab10 in Fig 7B could be shown to evaluate alternatives: first, that Rab11 and apically secreted Cad99C follow the same trans-Golgi to PM route but in different carriers; second, that transmembrane Cad99C is not secreted but just recycled through Rab11 once secreted.

The published YFP-Rab11 is too dim to track in fast, live imaging experiments and we cannot therefore determine whether it moves with Cad99c from the Golgi to the apical Rab11^+ve^ recycling endosomes, although in fixed samples it is clearly present on both (Figure 1G). We have now added co-localisation of Cad99c and Rab11 to the graph in Figure 1G, as suggested by the reviewer, and observe strong co-localisation that peaks at 20', before the peak of Cad99c co-localisation with the TGN marker Golgin-245 (22'), followed by a second peak of Cad99c co-localisation with Rab11 at 25'. This is consistent with the movement of Cad99c in Rab11 +ve vesicles from the Golgi to the apical recycling endosomes. However, the main argument that Cad99c traffics through Rab11^+ve^ recycling endosomes on its way to the apical plasma membrane is that Cad99c co-localises with Rab11 in sub-apical vesicles before any appreciable amount of protein has reached the apical plasma membrane. It therefore cannot have been exocytosed and then endocytosed to reach this compartment. Indeed, this is the whole point of using the RUSH system, which allows one to visualise the first passage of the cargo from the ER to the plasma membrane without the confounding effects of endocytic trafficking in the opposite direction.

 
3. In Fig. 7D-F it is shown that a Nidogen wave shifts Rab10 distribution bias from trailing to leading. This is difficult to understand considering that Collagen IV, secreted to trailing edge, is also transported in Rab10 vesicles. How would the motors or the microtubules know what the Rab10 vesicle contains? Does the Nidogen wave cause Collagen IV mistargeting to leading edge or are they sorted into different Rab10+ populations? Is it possible that the wave itself (not the fact that it's Ndg) somehow causes the shift? Would a collagen wave do the same (change direction to leading)? I understand the experiments to address these questions may be difficult to design, but the title points to this differential planar targeting as the main experimental finding in the paper. At least, the authors should check that endogenous Ndg (protein trap, not UAS or UAS wave) is secreted towards the leading edge in normal conditions.

As we mentioned in our response to referee 1, we have attempted to image the trafficking of endogenous Nidogen labelled by a protein trap insertion, but it was too dim to detect in movies, nor could we see the secreted protein at the lateral sides of the cells because it diffused away too rapidly. We were also unsuccessful in our attempt to examine whether the over-expression of Nidogen affects the direction of Collagen IV secretion. Our claim that Nidogen and Collagen IV are secreted from opposite sides of the follicle cells therefore rests on imaging them in different ways, Nidogen by tracking a pulse of over-expressed protein released from the ER and Collagen IV, by visualising the endogenous protein accumulating laterally between expressing and non-expressing follicle cells. We do not see this as a problem, because we do not share that reviewer's concern that the over-expression of Nidogen is somehow switching the direction of its basal trafficking. Firstly, the other cargoes that we have examined (Cadherin99c, E-cadherin, Fasciculin3) are secreted in the same place, whether or not they are over-expressed. Secondly, we do not think that Rab10 directs the planar-polarised trafficking of Nidogen and Collagen IV at the basal side of the cell. There is convincing evidence from Zajac and Horne-Badovinac (2023) that Rab10 directs the transport of ECM-containing vesicles to the basal side of the cell, and it would be unusual for the same Rab protein to mediate two distinct transport steps. We therefore favour the view that the planar-polarised trafficking is under the control of other adaptor proteins, which might be Rabs and their effectors, and these will be different for Nidogen and Collagen IV. If this is the case, Rab10 would be a passenger on at least two distinct populations of basal vesicles, one that moves towards the trailing edge and another that moves towards the leading edge. The wave of Nidogen produced by the RUSH system increases the population of Rab10^+ve^ vesicles moving towards the leading edge without affecting the population moving towards the trailing edge, resulting in the shift in the directional bias of Rab10^+ve^ vesicle movements. We now explain this interpretation in the discussion as follows:

" *Rab10 is required for the Khc73-dependent transport of ECM vesicles along microtubules to the basal side of the cell [20] . Once these Rab10^+ve^ vesicles reach the basal side, it seems likely that other adaptor proteins take over from Rab10 to mediate the planar-polarised trafficking of Collagen IV and Nidogen vesicles in opposite directions. In this scenario, Rab10 would be a passenger on two distinct populations of basal vesicles, one containing Collagen IV that moves towards the trailing edge and another containing Nidogen that moves towards the leading edge. This would explain the shift in the direction of Rab10^+ve^ vesicle movements on Nidogen over expression. The wave of Nidogen produced by the RUSH system will increase the population of Rab10^+ve^ vesicles moving towards the leading edge, without affecting the population moving towards the trailing edge, thereby reversing the directional bias.*"

4. The authors should comment on the fact that Cad99C is basolaterally mistargeted upon microtubule cytoskeleton disruption, which is in contrast with Khanal et al., 2016, who reported basolateral accumulation of unsecreted Cad99C (endogenous). The authors state that a low level of basolateral leakage for UAS-SBP-Cad99C already occurs without disrupting microtubules (Fig 1C, 2nd paragraph of results), opening the possibility that mistargeting would not occur with the endogenous protein or at endogenous levels.

The difference between the results of Khanal et al and our experiments are that they removed all dynein activity and looked at the steady state distribution of Cad99c, whereas we slowed dynein down and looked at the effects on Cad99c trafficking from the ER to the plasma membrane. When we treat cells with the dynein inhibitor, Ciliobrevin, we see all Cad99c retained in the Golgi. Thus, the most likely explanation for the difference between our results and those of Khanal et al. is that dynein is required for the exit of Cad99c from the Golgi, but this function is not impaired by the Dhc^8-1^ mutant. We have added this to the text as follows: "*The observation that the slow Dynein mutant leads to the secretion of Cad99c at the lateral membrane of the follicle cells differs from the results of Khanal et al. (2018), who found that knock-down of dynein led to the intracellular retention of Cad99c. This disparity is most probably due to the difference between removing most dynein activity by RNAi-mediated knock-down and just slowing the speed of the motor. Indeed, treating follicle cells with the dynein inhibitor Ciliobrevin prevents the exit of Cad99c from the Golgi, indicating that dynein is required for Cad99c trafficking to the plasma membrane (data not shown). By contrast, Cad99c can exit the Golgi in Dhc^8-1^/ +, but is no longer efficiently transported apically, allowing its lateral secretion*."

This is an important point because it is used to argue that directionality of microtubule-based transport is enough to explain apical targeting (sufficiency of directionality to explain basal targeting was already demonstrated by the kinesin mutations that mistarget collagen IV to the apical side in Zajac 2022).

We agree that the results of Zajac et al are relevant to this point and have now included this in the discussion. However, they find that knock down of Khc73 leads to the secretion of Collagen IV at the top of the lateral domain. They do see an apical accumulation of Collagen IV when both kinesin and Khc-73 are knocked down, but it is hard to tell whether this signal is extracellular or in a subapical membrane compartment, as it seems to be slightly below the aPKC staining, which is inside the cell. In support of the latter view, Rab10, which is not a secreted protein, also accumulates apically in these double knockdown cells.

Minor points:

A. Fig 2G,H: It may be worth pointing out that the Cad99C transmembrane cargo seems to take longer to traverse the Golgi than soluble Ndg. The authors could discuss this in the context of intra-Golgi transport models and compare with speed figures in previous literature.

We are happy to point this out, but don't understand what the significance of this might be to the debate between the cisternal maturation, anterograde trafficking and the rapid partitioning models. We would prefer not to wade into this controversy and just point out the relevant observations. We have therefore added the following text to the results: " *Nidogen also transits the Golgi in < 4 minutes, which is much faster than Cad99c. Soluble cargoes have been observed to transit the Golgi more quickly than membrane-spanning cargoes in a variety of contexts, but the reason for this difference is still a subject of debate [29–32]."*

B. In Fig 2G,H, Ndg takes longer to traffic from the trans-Golgi to the PM than Cad99C. Is this explained by the differential apical and basal microtubule-based routes the two cargos follow? Their different speeds or displacement lengths? Again, perhaps this is in the data already.

Nidogen starts to appear basally 6 minutes after biotin addition but accumulates extracellularly more slowly that Cad99c (i.e. the slope of the line showing its co-localisation with WGA is shallower than that for Cad99c and CellMask). We think that this difference arises because only some of the over-expressed Nidogen binds to the adjacent ECM and the rest diffuses away. In support of this view, we have co-cultured "Nidogen RUSH" egg chambers with wildtype egg chambers and observed that the labelled Nidogen binds to the ECM of the wildtype egg chambers in the same dish.

C. It is stated that Collagen IV, because it is a dimer (actually, an obligate heterotrimer of the two Collagen IV chains), is not amenable to the RUSH approach. I wonder if this is based on speculation or actual attempts. I see in principle no problem if the hook is located in a place on either chain that does not affect trimerization. The other chain could be imaged instead of the hooked/overexpressed one to avoid imaging a mixture of trimer and excess monomer.

We attempted to perform RUSH on Collagen 25C, which forms trimers with Viking to form mature Collagen IV, using a UAS-SBP-SNAP- Collagen 25C transgene (a.k.a Col4a1). Unfortunately, SBP-SNAP- Collagen 25C remained stuck in the ER after the addition of biotin. We presume that this either because the SBP and SNAP tags disrupt is folding or because there is insufficient Viking for it to assemble correctly into trimers. We did not have time to generate the stocks to try over-expressing both SBP-Collagen 25C and Viking.

D. The fact that UAS-SBP-Ndg localizes to basement membrane should be shown (data not shown).

We have added this as Figure 1D.

E. I did not understand the experiment in 7 G,H. Was it in doubt that Collagen IV is secreted to the trailing edge of cells (Zajac 2022)?

Zajac et al. tracked Rab10^+ve^ vesicles rather than Collagen IV itself, which is too faint to follow in fast movies. Since our results suggest that there is more than one population of basal Rab10^+ve^ vesicles, we felt that using Rab10 as a proxy for Collagen IV could cause problems. We therefore needed to prove that Collagen IV is indeed secreted at the trailing edge.

F. The fact that colcemid completely inhibits Ndg transport perhaps deserves some discussion.
We state in the results describing the effects of colchicine treatment on Nidogen that: "*Indeed, most Nidogen appeared to remain in the Golgi, as shown by its co-localisation with Golgin245, suggesting that the exit of Nidogen from the TGN requires the pulling forces exerted by microtubule motor proteins."* We don't have anything more to say about this, but could move this to the discussion if necessary.


Reviewer #3: In this article, Richens et al. investigate the tracking of Cadherin 99C and Nidogen during their secretion using the RUSH system. They achieve this by 1) developing a new tracking tool (MSP-tracker and viewer) and 2) employing the tool to study the secretion of RUSH cargoes. This manuscript is an interesting mix of image analysis development and biological experiments. My two main feedback on this manuscript are:
The biological observations remain limited at this stage. What is tracked is also unclear (see below). Clarification on the tracking and imaging conditions (3D and speed) is essential, and I cannot provide any recommendations without them.
The tracking tool developed could be better documented.
The methods section is poorly documented, making the data hard to evaluate. A more detailed and comprehensive account of the data would be beneficial.
The authors could consider whether Methods & Resources Articles would be a more fitting format for communicating their findings. As the work can be clearly divided into tool development and biological discoveries, I will provide feedback on these two categories.

Analyses of cargo secretion:
The authors appear to claim that they successfully tracked individual vesicles using 2D imaging with a confocal microscope, capturing one frame every 5 minutes. However, tracking vesicles in the cell volume under these conditions is impossible. Fast 3D imaging (on a second-time scale) and 3D tracking would be required to track individual vesicles. The authors need to clarify how these experiments were performed and analyzed.

This reviewer seems to have overlooked large sections of our manuscript and supplementary material that describe how we made the movies and document how to download and use MSP-tracker and viewer.

Most movies used for tracking were taken on the Zeiss Airyscan in fast mode at 2.3-4.5 frames/second, with a few on a spinning disc microscope at 2 frames/ sec. This information was included in the Movie legends: *" e.g. Movie 5: Cadherin99c RUSH – All movies were taken on the Zeiss Airyscan using Airyscan fast mode at 4.5 frames per second.",* and is also shown within the movies in the title before each movie section.

To make this even clearer, we have amended the relevant section of the main text to : "*To examine the movement of post-Golgi carriers from the TGN to the plasma membrane, we performed fast time-lapse imaging* ***at 2 - 4.5 frames/second*** *of labelled cargoes 15-20 minutes after the addition of biotin, i.e. just after their peak accumulation in the TGN."*

All our movies are 2D, because one needs a frame rate of at least 2 frames/second to follow vesicles that move at speeds >1µm/second. This precludes collecting multiple sections for each time point.

The supplementary videos are pixelated and show a large drift.

The only movie that showed a large drift was movie 1, although this did not prevent the viewer from seeing the apical secretion of Cad99c. Nevertheless, we have now replaced this movie with another that does not show significant drift. The drift in the other movies is insignificant, and we ask the reviewer to bear in mind that we are not imaging cultured cells adhered to a coverslip, but living ovaries ex vivo. This comes with the unavoidable movements of the egg chambers down the ovariole due to the peristalsis of any remaining muscle sheath, and the simultaneous rotation of the egg chambers around their long axes. This is most obvious in the long movies showing the time course of secretion.

In addition, there are no video examples of successful tracking of individual vesicles after registration. Without convincing evidence of successful analyses, it is impossible to evaluate the biological findings further.

The reviewer seems to have overlooked the 12 movies showing the successful tracking of individual vesicles in Movies 5,6,8 and 9. In each case, we show the movie in one half of the screen and the same movie with the tracks overlaid in the other half.

MSP-tracker and viewer:Kudos to the authors for sharing their code. The GitHub repo has minimal documentation, though, and only a few users will be able to use this tool out of the box. I would not recommend that my lab use this tool at this stage.

The reviewer appears to have missed the 20 page user manual on github (now moved to https://doi.org/10.17863/CAM.114339), which we have now supplemented with two instructional videos

I could not find a test dataset associated with the software. Including a test dataset would allow users to better understand and evaluate the tool.

The training dataset is available at <https://doi.org/10.17863/CAM.114338>

A video showing the software in action would be beneficial for users to grasp its functionality and application.

We have also now added two instructional videos that show how to open and use MSP-tracker and MSP-viewer at <https://doi.org/10.17863/CAM.114339>

In the manuscript, the rationale for developing a new tracking tool for this particular application could be better explained. If the main issue is signal-to-noise, why not include denoising in the analysis pipeline?

As we state in the text "*The particle detection module consists of background subtraction step followed by the identification of spots of different sizes and brightness using a multi scale spot enhancing filter (MSSEF)*", which is equivalent to denoising. However, the main issue for successful tracking is distinguishing the vesicles from other labelled vesicular structures in the images, such as the Golgi, which are larger and brighter. This is why the convolutional neural network is key, as the system is trained to recognise the vesicles of interest.

While the authors effectively assess their software on synthetic and real data, the comparison with other tracking tools (TrackMate and SpotTracking) lacks any methodology. Given the numerous options available in TrackMate and SpotTracking, it is crucial to provide information on how these comparisons were conducted. In addition, a comprehensive parameter sweep is necessary for a fair comparison.

Parameters for each software (MSP-tracker, TrackMate and SpotTracking) were set individually for each video-sequence to achieve the best performance and we now state this in the text: " *Parameters were set individually for each video sequence to achieve the best performance in each software."*

The author indicates, "MSP-tracker performed much better than the other programs on this metric, as it detected nearly 90% of the annotated tracks compared to 40% for Trackmate and SpotTracking (Figure 4C)". But isn't the MSP tracker trained to detect the correct spots using manual annotations? What detectors were used in TrackMate? For instance, would training a Stardist/ilastik model trained to detect the spots perform as well? Is the issue in the tracking or the detection of the spots to start with?

The main challenge in vesicle tracking is the detection of spots, and training on specific data allowed us to get better results. The advantage of the proposed method is the relaxed requirement for data annotation, where not every particle has to be annotated for each frame.

While Stardist is very suitable for cell tracking, it is not useful for the tracking of vesicles, which have less distinct boundaries and smaller sizes.

The manuscript lacks references to the vast ecosystem of other available tracking tools, especially other tracking tools evolving in a similar space (i.e., tools available in Python)
The manuscript lacks information on the limitations of the tool and possible future developments. Is 3D tracking possible?

We reference many other tracking tools, but limited our discussion to those that have a graphical user interface. As far as we are aware, we have mentioned all of these.

Since we can only collect data in 2D, we have not considered whether the software could be adapted to work in 3D.


Reviewer #4: The authors studied trafficking of vesicles to examine polarized secretion in epithelial cells using the RUSH system [14, 15]. A vesicle tracking software, MSP-tracker and MSP-viewer, was developed to quantify the dynamics of trafficking from the generated time-lapse movies.

MSP-tracker is an extended version of the authors previous work [45] and uses deep learning. Vesicles are detected by the multi scale spot enhancing filter (MSSEF) [46], and then a CNN classifier is employed for candidate vesicle selection to improve the detection efficiency. Pretrained models for the CNN classifier are available in the MSP-tracker, and the classifier can also be trained on new data. Tracking is performed by a two-step linking approach using tracklets and a connectivity score computed by a Bayesian network (BN). The assignment problem is solved by the Hungarian algorithm. From the trajectories, different measures are computed to quantify trafficking (e.g., distance, total time, orientation).

The developed tracking method was evaluated using vesicle data from the Particle Tracking Challenge (CTC) [Chenouard 2014] and real data of vesicles from the RUSH system. For the CTC data, the performance falls within the mid/upper range of published results. For the vesicle data from the RUSH system, the method outperforms two other software, Trackmate [23,32] and SpotTracking [49].

The developed interactive tracking software was applied to quantify trafficking of post-Golgi vesicles in time-lapse movies from the RUSH system. The results reveal that Cadherin 99C vesicles move apically which suggests that microtubule organization plays an important role in the secretion process. It also turns out that Nidogen vesicles undergo planar-polarised movement.

The manuscript is well written, the developed software outperforms other software, and relevant biological findings were obtained. I suggest revising the manuscript and addressing the following points:

1. More information on the architecture of the used Bayesian network (BN) should be provided.

We have now added the following text to the Materials and Methods: "*A Bayesian network (BN) is used to compute the connectivity score for each pair of the tracklets. This probabilistic graphical model is used to represent a set of discrete variables and their conditional dependencies and is implemented using pgmpy library*[14,15]" 

2. In the literature review on object tracking the authors mention the references [17-27,30-32]. More recent deep learning methods for particle tracking also applied to CTC vesicle data should be included.

We have added the following text to Results section: " *Many different tracking algorithms have been developed in recent years that use deep learning (DL) methods to enhance target detection and object tracking* [16–29]*. However, these machine learning methods usually require an extensive training dataset that must be updated for each new experimental system, which can be impractical and time consuming, some were developed for particular imaging techniques, while others perform poorly in a noisy environment. DL methods have also been integrated into several tracking platforms* [30] *, including TrackMate* [25]  *DeepTree* [31] *, ELEPHANT* [32]  *and others, but these are mainly designed for cell segmentation, rather than tracking small fast-moving particles. To address these drawbacks, we have developed an MSP (Multi-Scale Particle) framework to facilitate particle tracking in a noisy environment.*"

3. Fig 4, "coloured areas refer to the range of the results": More information should be given how "range of the results" is defined.

The range of the results refers to the interval between the lowest and the highest metric value for all the methods presented in the paper. The colouring of the area allows us to visualise the range without overwhelming the figure with additional information. We have modified the legend to Figure 4A as follows "*The lines represent the MSP-tracker results and coloured areas refer to the range between the highest and lowest scores in the original challenge paper* [19]*.*"

4. Fig 4, "MSP-tracker performs extremely well for the Jaccard similarity coefficient for the entire tracks, JSCθ": The term "extremely well" should be relaxed.

Done

5. "[20]The trafficking" and "[Chenouard2014]" should be corrected.

Done

1. Clark A, Meignin C, Davis I. A Dynein-dependent shortcut rapidly delivers axis determination transcripts into the Drosophila oocyte. Development (Cambridge, England). 2007;134: 1955–1965. doi:10.1242/dev.02832

2. Denef N, Chen Y, Weeks SD, Barcelo G, Schüpbach T. Crag regulates epithelial architecture and polarized deposition of basement membrane proteins in Drosophila. Developmental Cell. 2008;14: 354–364. doi:10.1016/j.devcel.2007.12.012

3. Lerner DW, McCoy D, Isabella AJ, Mahowald AP, Gerlach GF, Chaudhry TA, et al. A Rab10-Dependent Mechanism for Polarized Basement Membrane Secretion during Organ Morphogenesis. Developmental Cell. 2013;24: 159–168. doi:10.1016/j.devcel.2012.12.005

4. Zajac AL, Horne-Badovinac S. Kinesin-directed secretion of basement membrane proteins to a subdomain of the basolateral surface in Drosophila epithelial cells. Curr Biol. 2022;32: 735-748.e10. doi:10.1016/j.cub.2021.12.025

5. Viktorinová I, Dahmann C. Microtubule Polarity Predicts Direction of Egg Chamber Rotation in Drosophila. Curr Biol. 2013;23: 1472–1477. doi:10.1016/j.cub.2013.06.014

6. Karagöz GE, Acosta-Alvear D, Walter P. The Unfolded Protein Response: Detecting and Responding to Fluctuations in the Protein-Folding Capacity of the Endoplasmic Reticulum. Csh Perspect Biol. 2019;11: a033886. doi:10.1101/cshperspect.a033886

7. Doerflinger H, Benton R, Shulman JM, Johnston DS. The role of PAR-1 in regulating the polarised microtubule cytoskeleton in the Drosophila follicular epithelium. Development (Cambridge, England). 2003;130: 3965–3975. doi:10.1242/dev.00616

8. Nashchekin D, Fernandes AR, Johnston DS. Patronin/Shot Cortical Foci Assemble the Noncentrosomal Microtubule Array that Specifies the Drosophila Anterior-Posterior Axis. Developmental Cell. 2016;38: 61–72. doi:10.1016/j.devcel.2016.06.010

9. Khanal I, Elbediwy A, Loza MDCD de la, Fletcher GC, Thompson BJ. Shot and Patronin polarise microtubules to direct membrane traffic and biogenesis of microvilli in epithelia. Journal of Cell Science. 2016;129: 2651–2659. doi:10.1242/jcs.189076

10. Beznoussenko GV, Parashuraman S, Rizzo R, Polishchuk R, Martella O, Giandomenico DD, et al. Transport of soluble proteins through the Golgi occurs by diffusion via continuities across cisternae. eLife. 2014;3: e02009. doi:10.7554/elife.02009

11. Casler JC, Papanikou E, Barrero JJ, Glick BS. Maturation-driven transport and AP-1–dependent recycling of a secretory cargo in the Golgi. J Cell Biol. 2019;218: 1582–1601. doi:10.1083/jcb.201807195

12. Sun X, Tie HC, Chen B, Lu L. Glycans function as a Golgi export signal to promote the constitutive exocytic trafficking. J Biol Chem. 2020;295: 14750–14762. doi:10.1074/jbc.ra120.014476

13. Tie HC, Wang H, Mahajan D, Sun X, Chen B, Lu L. Quantitative intra-Golgi transport and organization data suggest the stable compartment nature of the Golgi. 2024. doi:10.7554/elife.98582.1

14. Ankan A, Panda A. pgmpy: Probabilistic Graphical Models using Python. Proc 14th Python Sci Conf. 2015; 6–11. doi:10.25080/majora-7b98e3ed-001

15. Dmitrieva M, Zenner HL, Richens J, Johnston DS, Rittscher J. Protein Tracking By CNN-Based Candidate Pruning And Two-Step Linking With Bayesian Network. 2019 Ieee 29th Int Work Mach Learn Signal Process Mlsp. 2019;00: 1–6. doi:10.1109/mlsp.2019.8918873

16. Vallotton P, Oijen AM van, Whitchurch CB, Gelfand V, Yeo L, Tsiavaliaris G, et al. Diatrack particle tracking software: Review of applications and performance evaluation. Traffic. 2017;18: 840–852. doi:10.1111/tra.12530

17. Meijering E, Dzyubachyk O, Smal I. Chapter nine Methods for Cell and Particle Tracking. Methods Enzymol. 2012;504: 183–200. doi:10.1016/b978-0-12-391857-4.00009-4

18. Smal I, Yao Y, Galjart N, Meijering E. Facilitating Data Association In Particle Tracking Using Autoencoding And Score Matching. 2019 Ieee 16th Int Symposium Biomed Imaging Isbi 2019. 2019;00: 1523–1526. doi:10.1109/isbi.2019.8759418

19. Chenouard N, Smal I, Chaumont F de, Maška M, Sbalzarini IF, Gong Y, et al. Objective comparison of particle tracking methods. Nat Methods. 2014;11: 281–289. doi:10.1038/nmeth.2808

20. Smal I, Meijering E. Quantitative comparison of multiframe data association techniques for particle tracking in time-lapse fluorescence microscopy. Med Image Anal. 2015;24: 163–189. doi:10.1016/j.media.2015.06.006

21. Ritter C, Wollmann T, Lee J-Y, Imle A, Müller B, Fackler OT, et al. Data fusion and smoothing for probabilistic tracking of viral structures in fluorescence microscopy images. Med Image Anal. 2021;73: 102168. doi:10.1016/j.media.2021.102168

22. Tinevez J-Y, Perry N, Schindelin J, Hoopes GM, Reynolds GD, Laplantine E, et al. TrackMate: An open and extensible platform for single-particle tracking. Methods. 2017;115: 80–90. doi:10.1016/j.ymeth.2016.09.016

23. Jaqaman K, Loerke D, Mettlen M, Kuwata H, Grinstein S, Schmid SL, et al. Robust single particle tracking in live cell time-lapse sequences. Nat Methods. 2008;5: 695–702. doi:10.1038/nmeth.1237

24. Allan, Caswell DB;, Keim T, Wel NC; van der, Verweij CM;, W. R. soft-matter/trackpy: Trackpy v0.5.0. Zenodo. 2019. doi:10.5281/zenodo.4682814

25. Ershov D, Phan M-S, Pylvänäinen JW, Rigaud SU, Blanc LL, Charles-Orszag A, et al. TrackMate 7: integrating state-of-the-art segmentation algorithms into tracking pipelines. Nat Methods. 2022;19: 829–832. doi:10.1038/s41592-022-01507-1

26. Spilger DPTAT of P in FMIUDLR. Deep particle tracker: automatic tracking of particles in fluorescence microscopy images using deep learning. Deep Learning in Medical Image Analysis and Multimodal Learning for Clinical Decision Support. 2018.

27. Yao Y, Smal I, Grigoriev I, Akhmanova A, Meijering E. Deep-learning method for data association in particle tracking. Bioinformatics. 2020;36: 4935–4941. doi:10.1093/bioinformatics/btaa597

28. Newby JM, Schaefer AM, Lee PT, Forest MG, Lai SK. Convolutional neural networks automate detection for tracking of submicron-scale particles in 2D and 3D. Proc National Acad Sci. 2018;115: 9026–9031. doi:10.1073/pnas.1804420115

29. Wollmann T, Ritter C, Dohrke JN, Lee J-Y, Bartenschlager R, Rohr K. Detnet: Deep Neural Network For Particle Detection In Fluorescence Microscopy Images. 2019 Ieee 16th Int Symposium Biomed Imaging Isbi 2019. 2019;00: 517–520. doi:10.1109/isbi.2019.8759234

30. Pylvänäinen JW, Gómez-de-Mariscal E, Henriques R, Jacquemet G. Live-cell imaging in the deep learning era. Curr Opin Cell Biol. 2023;85: 102271. doi:10.1016/j.ceb.2023.102271

31. Ulicna K, Vallardi G, Charras G, Lowe AR. Automated Deep Lineage Tree Analysis Using a Bayesian Single Cell Tracking Approach. Front Comput Sci. 2021;3: 734559. doi:10.3389/fcomp.2021.734559

32. Sugawara K, Çevrim Ç, Averof M. Tracking cell lineages in 3D by incremental deep learning. eLife. 2022;11: e69380. doi:10.7554/elife.69380
