# Supplementary material for: MSP-tracker: A versatile vesicle tracking software tool used to reveal the spatial control of polarized secretion in Drosophila epithelial cells
Source: PLoS Biol. 2025 Apr 10;23(4):e3003099. doi: 10.1371/journal.pbio.3003099 (PMC12021295; doi:10.1371/journal.pbio.3003099)
Supplement: Attachment — Submitted filename: PBIOLOGY-D-24-01269_R2_reviewer.docx [file pbio.3003099.s027.docx]

**General Comments:** This revised manuscript addressed most of the concerns I raised in the first round, but several issues require attention for clarity, consistency, and scientific accuracy. I would recommend the authors conduct a thorough review of figure and movie references to ensure all are correctly labeled, referred to, and discussed in the text.

**1. Figure and Supplementary Material Issues:**

- **Figure 4B:** The label "Low" is missing the letter "L." Please ensure all figure labels are complete and accurate.
- **Supplementary Figures:**
  - There is inconsistent usage of terms such as "Supplementary figures" and "Fig. SXX" (e.g., **Fig. S8G**, p. 11).
  - **Fig. S8** is referred to between Supplementary Figures 2 and 3 but does not appear in the supplementary information. Please confirm whether this is a mis-reference and correct it accordingly.
  - **Supplementary Figure 5** appears identical to Supplementary Figure 3. Based on the legend for Supplementary Figure 5, it seems that the actual Supplementary Figure 5 may be missing. Therefore, I cannot judge any of the data referred to Supplementary Figure 5 throughout the manuscript. Please clarify and revise.
  - Panels A, C, E, G of **Supplementary Figure 6** are never mentioned in the manuscript. Please ensure that all figure panels are referenced and explained.
  - The results from **Supplementary Figure 6J and 6K** are neither mentioned nor discussed. Please include these results in the manuscript.
- **Movie 10:** On p. 17, there is a reference to dynein inhibition affecting Cad99c trafficking, with "data not shown" (“Indeed, treating follicle cells with the dynein inhibitor Ciliobrevin prevents the exit of Cad99c from the Golgi, indicating that dynein is required for Cad99c trafficking to the plasma membrane (data not shown)”). Is this data represented in Movie 10? If so, explicitly reference it in the text. Currently, Movie 10 is unreferenced in the text. Please either reference it explicitly or clarify its relevance.

**2. Specific Questions and Data Clarifications:**

- **Dhc8-1 Allele and Homozygous Clones:**
  - By how much does Dhc8-1 reduce dynein-dependent transport speed? Provide quantitative data or reference if available.
  - What is the nature of the Dhc8-1 allele? A brief description would enhance clarity.
  - Do homozygous clones for Dhc8-1 and Dhc6-6 show reduced velocity in basal-directed Cad99C transport?
- **Ndg Movement:**
  - What mechanism drives the apical movement of Nidogen (Ndg)? The authors state that “their trafficking either does not require dynein or is mediated by a form of the dynein motor complex whose speed is unaffected by the 8-1 mutation.” Could the authors elaborate on which alternative minus-end-directed motors might be involved in this process? Additionally, how could it be determined whether dynein is indeed contributing to the apical transport of Ndg? For instance, could this be assessed using *Dhc-RNAi* or similar experimental approaches?

**3. Terminology and Nomenclature of kinesin:**

- On p. 15, change "kinesin" to "kinesin-1" where appropriate (e.g., "This is significantly faster than kinesin").
- "Kinesin is the predominant motor that transports vesicles along basal microtubules" should also specify "kinesin-1."
- Please ensure to distinguish between kinesin-1 and kinesin-3 (Khc-73) throughout the manuscript.
