# Supplementary material for: MSP-tracker: A versatile vesicle tracking software tool used to reveal the spatial control of polarized secretion in Drosophila epithelial cells
Source: PLoS Biol. 2025 Apr 10;23(4):e3003099. doi: 10.1371/journal.pbio.3003099 (PMC12021295; doi:10.1371/journal.pbio.3003099)
Supplement: Attachment — Submitted filename: Response to the Reviewers.docx [file pbio.3003099.s029.docx]

**Response to the Editors**
I would be grateful if you could please tick ‘Methods and Resources’ as the article type in the online submission form upon resubmission (if this option is unavailable then I can also do this on your behalf). 
We have done this

In this case, we would suggest the following edit to the title. Please ensure you change both the manuscript file and the online submission system, as they need to match for final acceptance:

“MSP-tracker: a versatile vesicle tracking software tool used to reveal the spatial control of polarized secretion in Drosophila epithelial cells”

We have changed the title as requested

**Response to the referees**
**Reviewer #1:**
**General Comments:** This revised manuscript addressed most of the concerns I raised in the first round, but several issues require attention for clarity, consistency, and scientific accuracy. I would recommend the authors conduct a thorough review of figure and movie references to ensure all are correctly labeled, referred to, and discussed in the text.

**1. Figure and Supplementary Material Issues:**

- **Figure 4B:** The label "Low" is missing the letter "L." Please ensure all figure labels are complete and accurate.

Corrected

- **Supplementary Figures:**
  - There is inconsistent usage of terms such as "Supplementary figures" and "Fig. SXX" (e.g., **Fig. S8G**, p. 11).

Corrected

- **Fig. S8** is referred to between Supplementary Figures 2 and 3 but does not appear in the supplementary information. Please confirm whether this is a mis-reference and correct it accordingly.
- **Supplementary Figure 5** appears identical to Supplementary Figure 3. Based on the legend for Supplementary Figure 5, it seems that the actual Supplementary Figure 5 may be missing. Therefore, I cannot judge any of the data referred to Supplementary Figure 5 throughout the manuscript. Please clarify and revise.

We apologise for this, which was caused by mistakenly uploading the old version of Supplementary Figure 5

- Panels A, C, E, G of **Supplementary Figure 6** are never mentioned in the manuscript. Please ensure that all figure panels are referenced and explained.

These panels are now cited as follows: "This increase in directionality correlates with the increase in the abundance of long microtubules that extend along the apical-basal axis of the columnar follicle cells compared to the cuboidal cells at stage 8 (Supplementary Figure 7 A,C, E and G)" and " Colcemid treatment leads to the depolymerisation of almost all microtubules in the follicle cells, but a few stable microtubules persist in the apical region of the cells (Mock injected in Supplementary Figure 7A, C, E and G, colcemid treated in Supplementary Figure 7B, D, F and H )"

- The results from **Supplementary Figure 6J and 6K** are neither mentioned nor discussed. Please include these results in the manuscript.

These are now cited as follows" "We confirmed that this mutation does not affect the microtubule network by staining microtubules in WT and *Dhc*^8-1^/+ egg chambers (Supplementary Figure 7J and K)"

- Corrected
- **Movie 10:** On p. 17, there is a reference to dynein inhibition affecting Cad99c trafficking, with "data not shown" (“Indeed, treating follicle cells with the dynein inhibitor Ciliobrevin prevents the exit of Cad99c from the Golgi, indicating that dynein is required for Cad99c trafficking to the plasma membrane (data not shown)”). Is this data represented in Movie 10? If so, explicitly reference it in the text. Currently, Movie 10 is unreferenced in the text. Please either reference it explicitly or clarify its relevance.

We thank the reviewer for spotting this mistake. We now cite Movie 10 correctly

**2. Specific Questions and Data Clarifications:**

- **Dhc8-1 Allele and Homozygous Clones:**
  - By how much does Dhc8-1 reduce dynein-dependent transport speed? Provide quantitative data or reference if available.

We have now added both references that used this allele and have cited the speed reduction observed in each case: " We therefore used the MSP package to analyse Cad99c carrier movements in stage 8 and 9 follicle cells heterozygous for a dominant EMS-induced mutation in the Dynein heavy chain, *Dhc*^8-1^, which reduces the speed of the dynein-dependent transport of *gurken* and *bicoid* RNAs by 50% and 36% respectively [63–65]

63. Gepner J, Li M, Ludmann S, Kortas C, Boylan K, Iyadurai SJP, et al. Cytoplasmic Dynein Function Is Essential in Drosophila melanogaster. Genetics. 1996;142: 865–878. doi:10.1093/genetics/142.3.865

64. MacDougall N, Clark A, MacDougall E, Davis I. Drosophila gurken (TGFalpha) mRNA localizes as particles that move within the oocyte in two dynein-dependent steps. Dev Cell. 2003;4: 307–319. doi:10.1016/s1534-5807(03)00058-3

65. Trovisco V, Belaya K, Nashchekin D, Irion U, Sirinakis G, Butler R, et al. bicoid mRNA localises to theDrosophilaoocyte anterior by random Dynein-mediated transport and anchoring. eLife. 2016;5: e17537. doi:10.7554/elife.17537

- What is the nature of the Dhc8-1 allele? A brief description would enhance clarity.

The nature of this allele is not known

- Do homozygous clones for Dhc8-1 and Dhc6-6 show reduced velocity in basal-directed Cad99C transport?

We have now added the data showing that the basal speeds are unaffected to Supplementary Figure 6D

- **Ndg Movement:**
  - What mechanism drives the apical movement of Nidogen (Ndg)? The authors state that “their trafficking either does not require dynein or is mediated by a form of the dynein motor complex whose speed is unaffected by the 8-1 mutation.” Could the authors elaborate on which alternative minus-end-directed motors might be involved in this process? Additionally, how could it be determined whether dynein is indeed contributing to the apical transport of Ndg? For instance, could this be assessed using *Dhc-RNAi* or similar experimental approaches?

We don't know how Nidogen is transported apically and have given all the information that we possess. Dhc-RNAi is hard to interpret because complete knock-down of dynein blocks cell division, disrupts the morphology of the egg chambers and alters microtubule organisation. Since this is a now a Methods and Resources paper, we think that investigating this question further is beyond the scope of this work.

**3. Terminology and Nomenclature of kinesin:**

- On p. 15, change "kinesin" to "kinesin-1" where appropriate (e.g., "This is significantly faster than kinesin").
- "Kinesin is the predominant motor that transports vesicles along basal microtubules" should also specify "kinesin-1."
- Please ensure to distinguish between kinesin-1 and kinesin-3 (Khc-73) throughout the manuscript.

Corrected

**Reviewer #2:** In this revised version of the manuscript, Richens et al. have addressed satisfactorily most of the points I raised in my review. I only have two remaining comments:
  
- Point 1: I disagree that this is a tangential issue and strongly recommend that this result be included in the manuscript. Consider the alternative where Cad99C and Ndg are released from different ERES: in that case, where the two different ERES populations locate, within the cell and with respect to microtubule tracks, would need to be assessed before concluding differential post-Golgi trafficking. Showing that both cargos traffic from all ERES makes the strongest possible case that they are actively sorted and differentially transported post-Golgi. In addition, it is an important finding that two such different cargos (TM and soluble ECM) are equally collected in all ERES. It raises the biological significance of the paper.

We have now added this result as Supplementary Figure 3 and mention it in the text as follows: " Large ECM cargoes like Collagen IV have been found to leave the ER through specialised ER exit sites (ERES) [33]. We therefore tested whether this is also the case for Nidogen by co-expressing SBP-SNAP-Nidogen and SBP-HALO-Cad99c and determining whether Nidogen leaves the ER through distinct ERES from Cad99c. However, we observed that all ERES that are positive for Cad99c are also labelled by Nidogen, indicating that a soluble ECM cargo and an apical transmembrane protein exit the ER through the same sites (Supplementary Figure 3)."

- Point 3 (also point 3 in referee #1's review): It is unfortunate that the direction of endogenous Ndg secretion could not be determined. This would have strengthened the claim that Ndg and Col IV follow divergent basal routes. The technical reasons for this are understandable, but I would ask that the possibility that endogenous and overexpressed Ndg behave differently is acknowledged.

We acknowledge this possibility in the discussion as follows: " Nidogen vesicles show a clear bias in their planar-polarised basal movements towards the leading edge of the migrating follicle cells, indicating that most Nidogen is secreted from this side of the cell, **at least when over-expressed with the RUSH system**.


**Reviewer #4**: Most of my comments were addressed.

Ref 43 should be corrected ("DPTAT of P in FMIUDLR")

We have corrected this reference.
